# Laparoscopy in the Surgical Management of the Non-Palpable Testis

**DOI:** 10.3389/fped.2014.00028

**Published:** 2014-04-08

**Authors:** Javier Castillo-Ortiz, Luis Muñiz-Colon, Karina Escudero, Marcos Perez-Brayfield

**Affiliations:** ^1^Division of Urology, University of Puerto Rico, San Juan, PR, USA; ^2^Division of Urology, Inter-American Hospital for Advanced Medicine, Caguas, PR, USA

**Keywords:** non-palpable testis, laparoscopy, orchiopexy, cryptorchidism

## Abstract

**Introduction:** To demonstrate that laparoscopic intervention should be considered as the initial surgical approach in the management of the non-palpable testis (NPT).

**Methods:** From 2007 to 2011, 100 testicular units underwent same surgeon laparoscopic management for NPT. Diagnostic laparoscopy was performed in all NPT and intra-abdominal testes (IAT) were managed by laparoscopic orchiopexy if low, laparoscopic Fowler-Stephens technique if high, and laparoscopic orchiectomy if atrophic. Percutaneous access to the abdomen was performed in most cases and laparoscopic management was performed with three 5 mm ports. We compared patient’s age, race, pre/post-operative exam, pre-operative work up, and IAT location upon laparoscopic intervention with surgical outcome. Fisher’s exact test for two independent proportions was used for statistical analysis and reported our results.

**Results:** One hundred testicular units underwent diagnostic laparoscopy for NPT. All patients were from Puerto Rican descent. 55.0% were found to be intra-abdominal and were subdivided into groups according to surgical intervention. Mean post-operative follow-up was 24 months. Patients 24 months of age or younger undergoing diagnostic laparoscopy for NPT had a statistically significant probability of resulting in successful laparoscopic orchiopexy as opposed to laparoscopic orchiectomy due to an atrophied IAT (*n* = 55 testicular units, *p* < 0.05). No laparoscopic related complications were reported.

**Conclusion:** Our findings support the use of an initial laparoscopic approach in the NPT as the majority of these patients will have IAT, avoiding unnecessary inguinal and scrotal explorations. We also recommend that patients with IAT should undergo laparoscopic orchiopexy prior to 2 years of age to increase probability of successful management. Further studies focusing in patients with NPT are needed in the future to confirm our findings.

## Introduction

The undescended testis (UDT) is one the most common congenital abnormalities found in newborn males, affecting up to 4% of full term newborns and up to 45% of pre-term males (1). Of these, 20% of UDT’s are reported as being non-palpable testis (NPT) to the examiner (2). The NPT can be non-present, atrophic, or have a failure to descend and be found in a high scrotal, inguinal, or intra-abdominal location (IAT). Most series report that approximately 20–40% of non-palpable testes are intra-abdominal in location (3–8). There are well-established risk factors for UDT with the most frequently reported being low birth weight and short gestational period, along with the known associations of multiple congenital syndromes. More recently, theories have focused on environmental, geographical, and ethnic factors influencing the development of UDT (1).

Laparoscopic orchiopexy has become the preferred approach for the management of the NPT. To our knowledge, there are no reports on the incidence, diagnosis, and management of the NPT in the Puerto Rican population. We aim to demonstrate that laparoscopic intervention should be considered as the initial approach in the NPT.

## Materials and Methods

From August 2007 to November 2011, 100 testicular units underwent same surgeon diagnostic laparoscopy for NPT. Patients’ ages ranged from 5 to 144 months, with a mean age of 53.0 months. All patients were Puerto Rican in descent. Prior to surgical intervention, patients were carefully examined again after general anesthesia induction to confirm that the testes were non-palpable. Patients were placed in slight Trendelenburg position with rotation of the bed away from the affected side. Percutaneous access to the abdomen using the Veress needle technique was used in most patients (96% of patients). An umbilical 5 mm trocar was placed under direct vision, followed by two 5 mm trocars in standard laparoscopic fashion. Diagnostic laparoscopy was performed in all NPT. If testicular vessels were seen entering the internal inguinal ring, inguinal, or scrotal exploration was performed. If laparoscopy revealed an IAT, the testicle was classified as low (<2 cm from the IIR) or high (≥2 cm from the IIR); laparoscopic orchiopexy was performed if low, laparoscopic one- or two-staged Fowler-Stephens technique (FSO) if high, or laparoscopic orchiectomy if atrophic, as previously described by Papparella et al. (9). Laparoscopic orchiopexy was performed using the Prentiss maneuver in all cases. Orchiectomy specimens were sent for histological examination.

All patients were followed post-operatively at 3 weeks, 3 and 6 months thereafter for a mean post-operative follow-up of 24 months and findings noted included: surgical site infections, post-operative testicular location, and testicular size, measured at the time of surgery and compared to the normal contralateral testis on follow-up by a single surgeon. Complications were divided into intra-operative and long-term. We compared patients’ age, pre- and post-operative exam, pre-operative work up, and IAT location upon laparoscopic intervention with surgical outcome. Fisher’s exact test for two independent proportions was used for statistical analysis and our results reported. Institutional Review Board approval was obtained prior to the initiation of this study.

## Results

**Figure 1:**
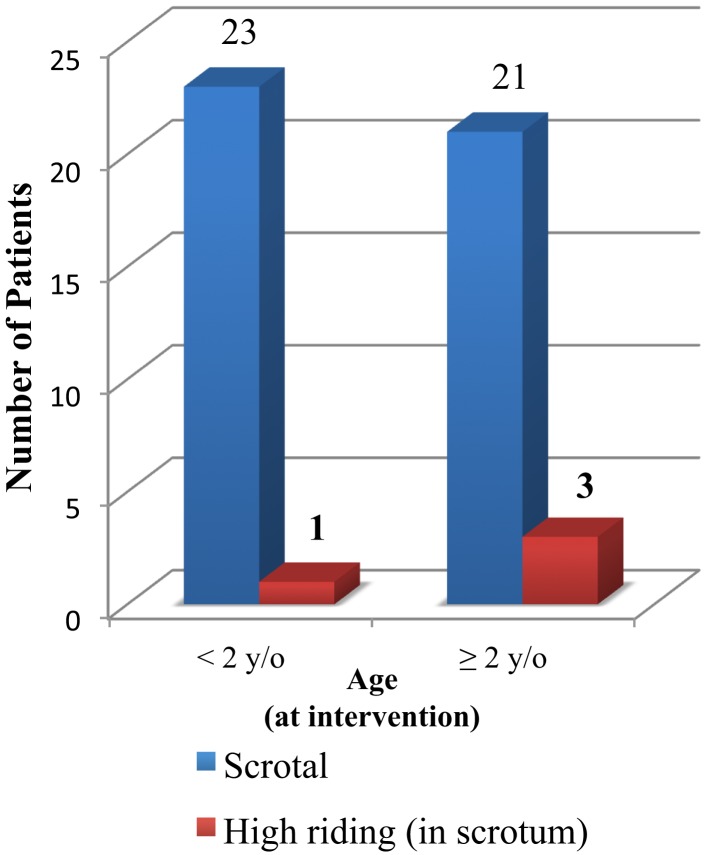
**Testis position after laparoscopic orchiopexy versus age (*p* = 0.61)**.

Diagnostic laparoscopy findings are presented on Table 1. Fifty-five testicular units (55.0%) were found to be intra-abdominal (48.9% right, 40.0% left, 11.1% bilateral) and were divided into two groups according to surgical intervention. Laparoscopic orchiectomy was performed in 7 testicular units due to intra-operative findings of an atrophied IAT (patient mean age: 84 months, range 24–144 months). Laparoscopic orchiopexy was performed in 48 testicular units (patient mean age: 23 months, range 5–84 months). Of the laparoscopic Fowler–Stephens Orchiopexies, one stage-FSO was applied to 6 testicular units (mean age 18 months, range 8–84 months), two-staged-FSO was applied to 1 testicular unit (first stage at 6 months; second stage at 12 months). Complications are shown on Table 2. We found 100% (48/48) of testicular units, which underwent laparoscopic orchiopexy presented with adequate size post-operatively; only one patient underwent two-staged-FSO and presented with adequate size post-operatively; 83.3% (5 out of 6) testicular units, which underwent one stage-FSO presented with adequate testicular size post-operatively. The latter patient, which underwent one stage-FSO, developed a scrotal wound infection post-operatively and subsequently developed left testicular atrophy on follow-up. High riding scrotal testes were found in four patients (one patient younger than 24 months and three patients with 24 months of age and older). Although it can be noted that a tendency exists in patients older than 24 months undergoing laparoscopic orchiopexy will result in a testis in a high riding scrotal position, this finding did not reach statistical significance (*n* = 48 testicular units, *p* = 0.61) (Figure 1). No intra-operative laparoscopic related complications were reported in our series and no complications relating to diagnostic laparoscopy were reported.

**Table 1:** **Findings upon diagnostic laparoscopy**.

Findings	Number of testis
Vessels/vas entering IIR*	38
Agenesis (no vessels identified)	3
IAT < 2 cm IIR	41
IAT > 2 cm IIR	7
Intra-abdominal blind-ending vessels	4
Atrophic IAT	7

*All patients with NPT and with vessels/vas entering the IIR on laparoscopy were found to be testicular nubbins and underwent orchiectomy through subsequent scrotal exploration.

**Table 2:** **Distribution of complication rates**.

	High riding	Infection	Atrophy	Total complication rate	Total success rate
Diagnostic laparoscopy	–	0	–	0% (0/45)	100% (45/45)
Laparoscopic orchiopexy	3	0	0	6.2% (3/48)	94% (45/48)
1FSO	1	1 (infection and atrophy in same patient)		33.3% (2/6)	67% (4/6)
2FSO	0	0	0	0% (0/1)	100% (1/1)
Total	7% (4/55)	1% (1/100)	1.8% (1/55)	5% (5/100)	95% (95/100)

**Figure 2:**
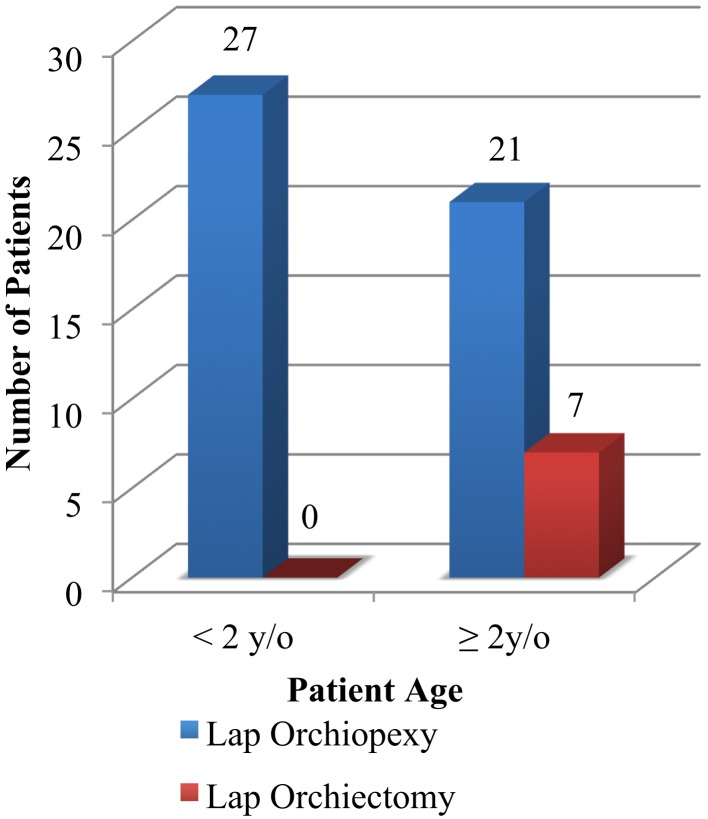
**Patient’s age versus laparoscopic intervention (*p* < 0.05)**.

Patients with 24 months of age or younger who underwent diagnostic laparoscopy for NPT had a statistically significant probability of resulting in successful laparoscopic orchiopexy as opposed to resulting in laparoscopic orchiectomy due to an atrophied IAT (*n* = 55 testicular units, *p* < 0.05) (Figure 2).

## Discussion

There is great variability on published literature on UDT regarding the incidence of IAT in NPT, in which most range from 20 to 40% (3–8). Genetics, geographical differences, environmental factors, race, and ethnicity have been proposed as possible factors affecting the rates of UDT. This variability on UDT rate may be in part explained by the heterogeneity of studied populations, that group genetic, ethnic, and geographically diverse populations in the same studied cohorts (1). In addition, a recent literature review on the frequency of UDT amongst boys from birth to adolescence demonstrated that only 6 of 46 (13%) of studies recorded ethnicity. The majority of the reported studies (40 of 46, 87%) did not take ethnicity into account (1). Our study groups a cohort of patients and found an increased incidence of IAT (55%) as compared to other published studies. As our studied population includes patients of similar racial background, one might suggest that race plays a significant role in the pathophysiology of the UDT.

Despite numerous publications regarding the management of the NPT, the debate on the proper initial surgical approach on this common urological condition continues. Even though the laparoscopic management has gained widespread approval over the last several years, opponents to this approach refer that the majority of NPT are extra-abdominal and can be managed through a scrotal or inguinal incision without exposing the patient to the risks of an unnecessary laparoscopic procedure (2–4, 9). The lower incidence of IAT in the literature is used by the proponents of a scrotal/inguinal approach to argue that up to 80% of laparoscopic interventions are unnecessary. As noted before, our incidence of IAT was higher than other published studies. This finding supports the use of an initial laparoscopic approach in our patient population as more than half of patients with NPT were found to be IAT (55%), avoiding scrotal or inguinal explorations in more than half of our studied population. Additionally, laparoscopic findings such as the absence of testicular vessels, which suggest testicular agenesis and intra-abdominal blind-ending vessels, avoided further abdominal, inguinal, and scrotal explorations in an additional 7% of patients. Taking this into consideration, initial laparoscopic intervention as a diagnostic tool in our patient population was beneficial to 62% of patients, as it provided relevant anatomic information that directed further surgical intervention. Similar results were demonstrated in other published data where diagnostic laparoscopy was beneficial to up to 73% of patients avoiding other unnecessary surgical approaches (9). These findings emphasize the decision to use laparoscopy as the initial surgical intervention in the NPT.

We also demonstrated that patients 24 months of age or younger undergoing diagnostic laparoscopy for NPT had a statistically significant probability of resulting in successful laparoscopic orchiopexy as opposed to resulting in laparoscopic orchiectomy due to an atrophied IAT. This finding gives importance to early diagnosis and treatment in the patient with UDT prior to 2 years of age. Spontaneous decent is felt to be complete in most patients by 6 months of age (10, 11). The above findings have let us to recommend treatment of the patient with NPT as early as 6 months but prior to 24 months of age.

Our series reported a 94% success rate in laparoscopic orchiopexy and an 85.7% success rate in laparoscopic FSO; results comparable to most published series and literature reviews, which report success rates ranging from 91.3 to 100% in laparoscopic orchiopexy (12–17) and 66.7 to 100% success in laparoscopic Fowler–Stephens orchiopexy (2, 12, 17, 18). We reported a 1% wound infection rate (1/100 laparoscopic interventions), attributed to a scrotal wound infection after a laparoscopic one-staged-FSO, a complication comparable to that of an inguinal or scrotal approach to the UDT, which report up to 3.3% of wound infections (19). We reported no intra-operative laparoscopic complications with almost all patients undergoing percutaneous access to the abdomen. Our data adds to recent studies that report no difference in complication rate between percutaneous and open Hassan technique in the pediatric population (20–22). Reports of complications regarding high riding testis after a single stage laparoscopic orchiopexy range from 0 to 8.7% (13, 17, 21, 23), consistent with a 6.2% complication rate in our studied population. High riding testis after FSO ranges from 0 to 7.4% in reported series (13, 17, 21); our series reports one patient with improper scrotal position after FSO resulting in 14.3%. Lastly, our data demonstrates an atrophy rate of 0 and 14.3% in laparoscopic orchiopexy and FSO groups, respectively; consistent with atrophy rates in the literature of 0–2.2 and 4.3–22.2% in laparoscopic orchiopexy and FSO groups, respectively (13, 17, 21, 24).

Several limitations are noted in our study. This is a retrospective cohort study based on a multi-institutional, single surgeon experience, possibly giving way to selection bias in our study. Even though testicular size was measured by a single surgeon in the operative setting and during follow-up and compared to the normal, contralateral testis for adequate size, this is a subjective appraisal of testicular size. We agree that testicular measurement by ultrasound offers a more precise and objective assessment and can be used as a confirmatory test to our clinical evaluation. Many of our patients are referred from other institutions after 12 months of age, which explains why our age group is higher than most published series and our elevated orchiectomy rate. In our series, only seven patients underwent Fowler–Stephens orchiopexy, and only one patient underwent a two-staged Fowler–Stephens orchiopexy, making it difficult to reach conclusion on this limited number of patients. Longer follow-up is needed to evaluate important outcomes such as infertility and risk of testicular malignancy later in life. Finally, to our knowledge, this is the first study, which has reported on the findings regarding the NPT in the Puerto Rican population.

In conclusion, our findings support the use of an initial laparoscopic approach in patients with NPT as the majority of these patients will have IAT, avoiding unnecessary inguinal and scrotal explorations. We also recommend that patients with IAT should undergo laparoscopic orchiopexy prior to 2 years of age to increase probability of successful management.

## Conflict of Interest Statement

The authors declare that the research was conducted in the absence of any commercial or financial relationships that could be construed as a potential conflict of interest.
